# “EspaiJove.net”- a school-based intervention programme to promote mental health and eradicate stigma in the adolescent population: study protocol for a cluster randomised controlled trial

**DOI:** 10.1186/s12889-018-5855-1

**Published:** 2018-07-31

**Authors:** Rocío Casañas, Victoria-Mailen Arfuch, Pere Castellví, Juan-José Gil, Maria Torres, Angela Pujol, Gemma Castells, Mercè Teixidó, Maria Teresa San-Emeterio, Hernán María Sampietro, Aleix Caussa, Jordi Alonso, Lluís Lalucat-Jo

**Affiliations:** 1 Research Department, Associació Centre Higiene Mental Les Corts, Grup CHM Salut Mental, C/ Numància, 103-105, Bajos, 08029 Barcelona, Spain; 2 Escola Superior Infermeria del Mar (ESIM), Universitat Pompeu Fabra (UPF), C/ Numància, 103-105, Bajos, 08029 Barcelona, Spain; 3 Departament of Clinical and Health Psychology, School of Psychology, Universitat Autònoma de Barcelona (UAB), Building B Campus de Bellaterra, 08193 Bellaterra (Cerdanyola del Vallès Barcelona), Barcelona, Spain; 4 Department of Psychology, Division of Clinical Psychology, Universidad de Jaen, Campus Las Lagunillas, s/n, 23071 Jaén, Spain; 5 Child and Juvenile Mental Health Centre of Les Corts, Associació Centre Higiene Mental Les Corts, Grup CHM Salut Mental, C/Montnegre 21, 3a planta, 08029 Barcelona, Spain; 6 Child and Juvenile Mental Health Centre of Sarria-Sant Gervasi, Associació Centre Higiene Mental Les Corts, Grup CHM Salut Mental, C/ Via Augusta 364-372, 4a planta, 08017 Barcelona, Spain; 7 Fundació Privada Centre Higiene Mental Les Corts, Grup CHM Salut Mental, C/ Numancia, 103-105, Bajos, 08029 Barcelona, Spain; 8 Activament Catalunya Associació, C/ Rocafort, 242 Bis, 3r B, 08029 Barcelona, Spain; 9 Spora Sinergies Consultoria social, C/ Floridablanca, 146, 08011 Barcelona, Spain; 10 Institut Hospital del Mar d’Investigacions Mèdiques (IMIM), C/ Dr. Aiguader, 88, 08003 Barcelona, Spain; 11 CIBER of Epidemiology and Public Health (CIBERESP) and Dept. Health and Experimental Sciences (DCEXS), Pompeu Fabra University (UPF), C/ Dr. Aiguader, 88, 08003 Barcelona, Spain

**Keywords:** Mental health, Promotion, Prevention, Stigma, Adolescence, School, Intervention, Mental health literacy

## Abstract

**Background:**

One half of adults who develop any mental disorder do so during adolescence. Previous literature showed that Mental Health Literacy (MHL) interventions impact mental health knowledge, reduce the associated stigma, and promote help-seeking among the adolescent population. However, evidence for the effectiveness and cost-effectiveness of these programmes remains inconclusive. The aim of this paper is to present a study protocol that evaluates the effectiveness of the “EspaiJove.net” programme. “EspaiJove.net” consists of a universal MHL intervention designed to promote mental health knowledge, increase help-seeking, reduce the stigma associated with mental illness, and prevent mental disorders in Spanish school settings.

**Methods:**

A school-based clustered randomised controlled trial (cRCT) design with 12 months of follow-up. **Subjects:** At least 408 secondary school students who attend the 3rd year of E.S.O (Compulsory secondary education for 13- to 14-year- olds) will be recruited from 8 schools within Barcelona city, Catalonia (Spain). **Intervention:** A dose-response intervention will be delivered with 4 arms: 1) Sensitivity Programme (SP) in Mental Health (1 h); 2) Mental Health Literacy (MHL) Programme (6 h); 3) MHL plus first-person Stigma Reduction (MHL + SR) (7 h); 4) Control group: waiting list.

**Primary outcomes:** 1) MHL: EspaiJove.net MHL Test (EMHLT); 2) Stigma: Reported and Intended Behaviour Scale (RIBS) and Community Attitudes toward the Mentally Ill (CAMI). **Others outcomes:** 1) Acceptability of intervention; 2) Mental health symptoms and emotional well-being (SDQ); 3) States of Change Scale (SCS); 4) Bullying and Cyberbullying; 5) Quality of life (EQ-5D); 6) Help seeking and use of treatment; 7) Health benefits.

**Discussion:**

Results would be informative for efforts to prevent mental disorders and promote mental wellbeing in secondary school students.

**Trial registration:**

NCT03215654 (date registration July 12, 2017).

**Electronic supplementary material:**

The online version of this article (10.1186/s12889-018-5855-1) contains supplementary material, which is available to authorized users.

## Background

The World Health Organisation (WHO) recognises mental disorders as one of the major health problems worldwide and as one of the biggest contributors to the global burden of disease [1]. With respect to the prevalence of mental disorders among adolescents and young adults, anxiety and depression are the most prevalent problems, there being a 12-month prevalence of 8% for anxiety and 4–5% for depression [2–4].

Mental disorders are a serious public health problem. It is estimated that 75% of all people suffering from a mental health disorder experienced onset before the age of 25 [5, 6], and 50% during adolescence [7]. Therefore, it coincides with the person’s crucial evolutionary moments which could represent a significant problem for the biological, psychological and social development of adolescents and young people.

Unfortunately, the prevalence of depression in adolescents and young adults has increased in the last few years, with a rise in the number of untreated young people [8]. Furthermore, young people delay their first visit to a health professional when suffering of a mental disorder. There is evidence that stigma and a lack of knowledge have been associated with a chronic delay in help-seeking [9, 10].

A systematic review of barriers and facilitators for help-seeking in young people found that one of the key barriers was stigma [11]. Stigma has a disabling impact on the individual’s sense of self, including diminished self-esteem, self-value and confidence [12]. Studies have shown a link between stigma, not seeking help and non-adherence to treatments [13].

A.F. Jorm introduced the concept of “Mental Health Literacy” (MHL) [14] in 1997, defined as a set of “knowledge and beliefs about mental disorders that aid recognition, management or prevention”. Therefore, MHL involves: a) the ability to recognise the development of mental disorders to facilitate early help-seeking behaviours; b) knowledge and beliefs about risk factors, the causes of mental disorders and how to prevent them; c) knowledge of how to get professional help, effective available treatments, and effective self-help strategies; d) attitudes that facilitate recognition and an adequate search for help; and e) knowledge and skills to provide first aid in mental health and support to other people. Also, the author proposed a training programme on mental health delivered to the general population to improve MHL interventions [9]. These studies point out the need to increase MHL due to the high number of mental disorder cases among young people and the low precocious diagnosis [15], the need to carry out these types of interventions in school settings [16–18] and the importance of working together in a coordinated way among the different services (educational centres, mental health services and community health) so as to benefit the health of young people [19].

Some MHL programmes for adolescents and young people have been developed in recent years in several countries [20–24]. These programmes suggest an improvement in mental health knowledge, the self-recognition of mental disorders among young people, and an improvement in facilitating monitoring in help-seeking and reducing mental health-related stigma immediately after an intervention [12, 13, 25–27]. However, recent systematic reviews and meta-analyses have shown that there is insufficient evidence about the positive impact of the school-based MHL interventions, and therefore more studies with better methodological designs are needed [28, 29].

The *EspaiJove.net**: a space for mental health* (EspaiJove.net) programme is a universal MHL programme which aims to promote mental health, prevent mental disorders, facilitate help-seeking behaviours and eradicate related stigma among secondary school students (between 11 and 18 years old) within the Spanish context. The programme integrates a multi-modal intervention that combines taught classes and training activities among schools with the use of Information and Communication Technology (ICT) such as the website www.espaijove.net and online consultation [30].

During the last five academic courses (2012/13 to 2016/17), 14,139 young people have been trained, of whom 7698 completed a questionnaire after the training activity. Despite there being widespread acceptability and satisfaction with the intervention (91% found it interesting and useful and 94% recommended the activity), a lack of robust evidence in MHL among the Spanish population and confirmation of good results meant that it was found that the EspaiJove.net programme needed to be evaluated.

In this article, we present a study protocol of a cluster randomised controlled trial that intends to evaluate the effectiveness of the universal school based-intervention “EspaiJove.net” programme. The main objective of the study is to evaluate whether the “EspaiJove.net” intervention has a short- (2 weeks) and long-term (6 and 12 months follow-up) impact on increasing MHL and reducing the related stigma among students who attend the 3rd year of E.S.O (Compulsory secondary education) or 9th grade (13- to 14-year- olds) in Barcelona, Spain. Furthermore, this study will assess the dose-response relationship for these outcomes. A secondary objective is to analyse the impact of the interventions on help-seeking behaviours, mental health symptoms and emotional well-being, bullying and cyber-bullying behaviours and health-related quality of life (HRQoL).

The primary hypothesis to be tested will be that: participants in the intervention groups (Sensitivity Programme (SP), MHL programme and MHL + SR programme) will increase their knowledge on mental health and mental disorders, and reduce the stigma associated with mental illness showing a dose-response relationship compared to a waiting list control group immediately after 2 weeks, 6 months and 12 months post-intervention.

A secondary hypothesis to be tested will be: participation in the intervention groups (SP, MHL and MHL + SR) will reduce mental health symptoms, increase emotional well-being and help-seeking behaviours, reduce bullying and cyberbullying at school, and improve HRQoL immediately after 2 weeks, 6 months and 12 months post-intervention, compared to a waiting list control group.

## Methods

### Study design

**Fig. 1:**
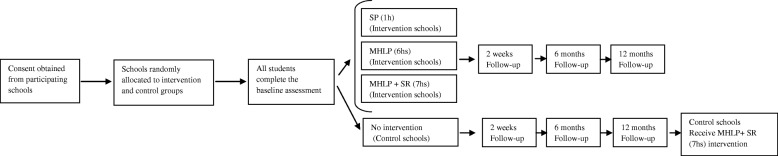
Schematic Illustration of the research design

The study is a multicentre and school-based clustered randomised controlled trial (cRCT) with four intervention arms. Eligible schools are randomly allocated either to an intervention group or a control group (see Fig. 1). Data will be collected at baseline, and at 2 weeks, 6 months and 12 months following the intervention. Each school will participate in the study for two academic years from September 2017 to September 2019.

The study has been designed in accordance with The Standard Protocol Items: Recommendations for Interventional Trials (SPIRIT) Statement for reporting a clinical trial protocol (see Additional file 1 for checklist) [31]. Trial registration NCT03215654.

### Sample

**Fig. 2:**
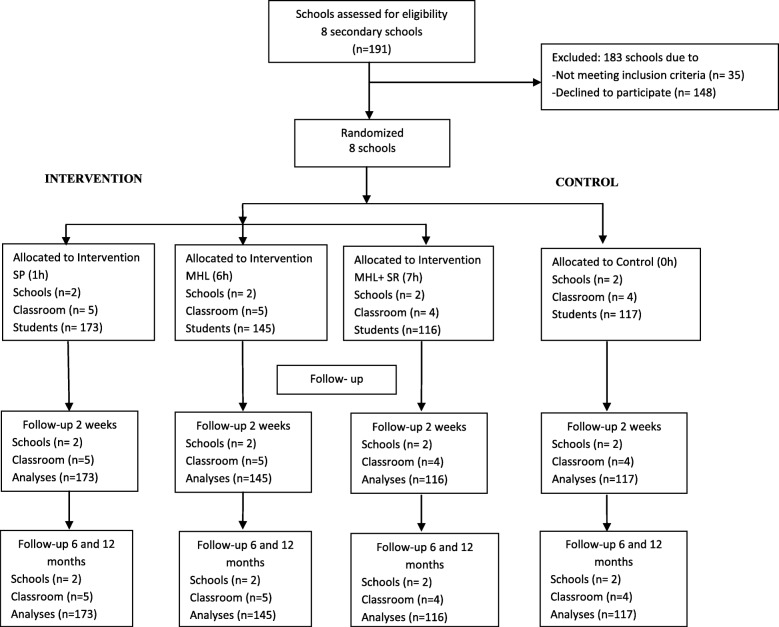
Flow chart of participants

Eligible participants are students who attend the 3rd year of E.S.O (Compulsory secondary education) or 9th grade (13- to 14-year- olds) who consent to participating in the study. Potentially eligible schools include 191 public and private schools located in 8 districts within Barcelona city, Spain. Of these, only those schools that agree to participate in research allow accessibility and commit to the continuity of the project will be involved. Each school will be distributed by the number of classes for each arm, and several randomisations will be conducted for each strata (up to five classes x grade) (see Fig 2). The inclusion criteria are as follows: adolescent students who (1) currently attend the 3rd year of E.S.O (Compulsory secondary education) in a public or private school located in Barcelona city, Spain; and (2) sign the informed consent. Exclusion criteria are as follows: students who (1) have attended a workshop by “EspaiJove.net” prior to the study; (2) attend any special education school; (3) have special educational needs attending any school; and (4) do not understand Spanish and Catalan.

### Sample size

A power analysis was conducted to determine the appropriate sample size. According to a previous RCT of MHL [32], assuming that we wanted to be able to identify a minimal difference of 0.18 effect size in MHL scores after the intervention. To detect differences in each group with one-way analysis of variance (ANOVA) that corresponds to a small effect size (0.18), with an alpha risk of 0.05, and a power of 80%, the adequate sample size required for this study in each group will be 85 students (*n* = 340). Assuming an attrition rate of 20% during the 12 months of follow-up after the intervention, we estimate that a total sample of 408 students will be required.

### Intervention

The *EspaiJove.net* programme started in 2010 and consists of: a) disseminating information on mental health; b) carrying out training activities with young people in schools; and c) carrying out training activities with professionals and families. The project is designed and delivered by professionals who are specialised in mental health and who move between different devices and educational centres to carry out training activities [30]. The programme includes education on several key aspects of mental health such as to: promote mental health wellbeing, facilitate help-seeking behaviours on their own, raise awareness of the consequences of risky behaviours, identify mental disorders and when/where to seek treatment, so as to eventually prevent and detect mental health-related problems early. Additionally, a person who has experienced mental illness first-hand will speak about his/her personal life experience with the students so as to aim to reduce any related stigma.

In this study we will asses three programmes (SP, MHL and MHL + SR) which consist of workshops run in the classrooms that cover the following topics:

- *Sensitivity programme (SP)* about mental health (1 h), including the concepts of mental health and mental disorders (emotional management).
- *Mental health literacy programme (MHL)* (6 h), including the contents of the EspaiJove.net programme. MHL programme module contents include: 1) Concepts of mental health and mental disorders. Mental health multidisciplinary team network; 2) Healthy and risky behaviours in mental health; 3) Social skills and antisocial behaviour, bullying and cyberbullying; 4) Anxiety, depression, self-harm, and suicidal behaviours; 5) Eating and behavioural disorders; 6) Substance abuse (alcohol and cannabis) and psychotic disorders.
- *Mental Health Literacy Programme plus Stigma Reduction* (*MHL + SR)* (7 h), including the six modules contained in the MHL programme and a presentation delivered by a person with first-hand experience of a mental disorder, accompanied by a voluntary member from the *Activament Catalunya Associació* (http://www.activament.org/es), a non-profit group specialised in reducing mental health-related stigma.

The intervention will be delivered by trained mental health nurses with vast experience in the treatment of children and adolescents. Nurses will receive previous training in relation to contents of the 6 modules of EspaiJove.net programme. The training period is 24 h (12 h theory and 12 h practice).

Each intervention (SP, MHL and MHL + SR) will be delivered to students during a tutorial class (one hour/week) and will be supported by Prezi-format presentations (https://prezi.com) about the 6 modules of the EspaiJove.net programme. Table 1 shows the contents of the EspaiJove.net programme among the different intervention groups.

**Table 1:** Contents of the EspaiJove.net programme in the different intervention groups

Hour	Workshop Contents	SP (1 h)	MHL (6 h)	MHL + SR (7 h)	Comparison group
1	Concepts of mental health (MH) and mental disorders (MD). Emotional management	x	x	x	Waiting List
2	Healthy and risk behaviours in MH		x	x	
3	Social skills and antisocial behaviours, bullying and cyberbullying		x	x	
4	Anxiety, depression and self-harm and suicidal behaviours		x	x	
5	Eating and behavioural disorders		x	x	
6	Substance abuse. Psychotic disorders		x	x	
7	Stigma Self-experience in MD and MH presentation of an activist of a voluntary member of Activament Catalunya Associació (http://www.activament.org/es)			x	

Abbreviations: *SP* Sensitivity Programme, *MHL* Mental Health Literacy Programme, *HML + SR* Mental Health Literacy Programme plus Stigma Reduction

Students from the control schools will act as a waiting list, and they will receive the MHLP +SR (7 h) programme after 12 months follow up, thus during the next academic year of 2018–2019.

### Recruitment process

The recruitment process for the trial began in September 2016. All eligible secondary schools within Barcelona city are contacted and encouraged to participate in the study. Emails providing information about the study are sent to all schools, and among those schools that had shown an interest in participating, a presentation is delivered at the school by two project researchers. Once a school agreed to participate, consent letters are sent out to the parents of all participating students.

Out of all the schools that accept participation in the study, a cluster randomisation by school and individual students as participants is conducted. The total sample estimated within a minimum of 408 adolescents will be divided into three experimental groups and one control waiting list. The randomisation assignment will be carried out through a computer program, and the group assignment will be 1:1:1:1.Each interviewer blinded the assessments.

Pre-treatment questionnaires will be completed approximately 2 weeks before the intervention by all students (intervention and control groups). A code will be used to match participant answers over time whilst preserving anonymity. One teacher will be the unique person responsible for keeping the identification number and corresponding student. Two researchers will be responsible for supplying each student with a questionnaire for its completion during each assessment within the study. The class teacher will be present in the classroom during the questionnaire administration involved in the data collection with the aim of minimising missing data and errors when providing the questionnaires.

A second evaluation will be performed approximately 2 weeks after the intervention. A third and fourth evaluation will be examined at 6 and 12 months, respectively. Table 2 shows the data collection and intervention programme timelines.

**Table 2:**
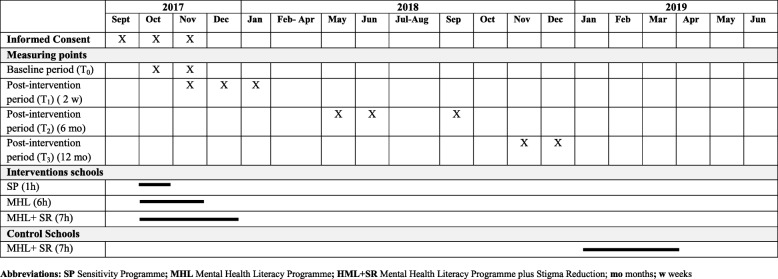
Data collection and intervention programme timeline

An Informed Consent Form (for children and parents) will be required for all participants prior to their involvement in the study. The confidentiality of the participants (children) will be protected using an encryption key for any personal details within the data (school 1–8, class 1–6, identification number 1–34). The key will be stored separately. Ethical approval was granted by the Fundació Unió Catalana Hospitals.

### Measures

The variables and instruments selected, as well as the evaluation programme timeline, are detailed in Table 3. Collected data will then be imported using a teleform format program onto a computer database.

**Table 3:** Outcome measures

Outcome measures	Timeline			
	Pre-intervention	Post-intervention		
		2 w	6 mo	12 mo
Primary outcomes				
1. Mental Health Knowledge				
EspaiJove.net Mental Health Literacy Questionnaire (EMHLQ)	X	X	X	X
2. Behaviour and Attitudes Towards Mental Health (Stigma)				
Reported and Intended Behaviour Scale (RIBS)	X	X	X	X
The Scaling Community Attitudes toward the Mentally III (CAMI) - Authoritarianism subscale	X	X	X	X
Others outcomes				
3. Acceptability and satisfaction				
Satisfaction questionnaire		X		
4. Mental Health Symptoms and Emotional Well-Being				
Strengths and Difficulties Questionnaire (SDQ)	X	X	X	X
5. Bullying and Cyber-Bullying Behaviours				
Bullying and Cyber-Bullying Questionnaire	X	X	X	X
6. Intentions to Change				
Intentions to Change Questionnaire (ICQ)	X	X	X	X
7. Health-related quality of life				
EuroQol Questionnaire (EQ-5D-5 L)	X	X	X	X
8. Help-seeking				
General Help-Seeking Questionnaire (GHSQ)	X		X	X
9. Use of Health Services and Treatment				
Use of Health Services and Treatment questionnaire	X		X	X
10. Health Benefits Questionnaire				
Familiar, academic and general health questions	X		X	X

*Abbreviations*: *mo* months, *w* weeks

First of all, socio-demographic variables will be collected: age; date of birth; gender; nationality; city/village; neighbourhood of residence or postcode. The following variables are listed below:

### Primary outcomes

The primary outcomes of this study are mental health literacy (MHL) and stigma associated with mental illness.

### Mental health knowledge

***EspaiJove Mental Health Literacy Test (EMHLT)*** is a self-reported questionnaire based on the thematic contents of the EspaiJove.net school programme. The EMHLT consist of 35 items, it uses two response formats: (i) the first part consists of a binary choice format (yes/no) for the recognition of mental disorders from a list of 15 different diseases; (ii) the second part contains 20 multiple choice questions with four possible answer options, in which only one is correct. To obtain the score for each individual in the test, the formula (A-E)/(n-1) is used, where A = the number of correct answers, E = the number of errors, and n = the number of options for each item. Thus, for section one of the test, the formula would be (A-E)/(2–1); and for the second part (A-E)/(4–1), where each correct answer adds one point to the total score. The total score of the test would be the result of the addition of both sections and would vary between 0 and 35 points. The cut-off point selected (pass test) was 25 points, representing approximately 70% of the answers [33].

### Stigma Behaviours and attitudes towards mental health

***Reported and Intended Behaviour Scale (RIBS)*** is used to assess reported and intended behavioural discrimination among the general public against people with mental health problems. The RIBS consist in 8 items; the first four items of the RIBS are designed to assess prevalence (past and current) of behaviour in each of the four contexts (1. living with; 2. working with; 3. living nearby; and 4.being in a relationship with someone with a mental health problem) while items 5–8 ask about intended (future) behaviour within the same contexts [34, 35]. For the purposes of this study, we selected four items from 5 to 8. It uses an ordinal Likert scale with five response options: “totally agree”, “somewhat agree”, “neither agree nor disagree”, “somewhat disagree”, “strongly disagree” from 5 to 1 points, respectively. The total score is obtained from a sum of the total answers ranging from 4 to 20. Higher scores indicate greater agreement with engaging the stated behaviour. The Spanish version will be used in this study.

***The Scaling Community Attitudes toward the Mentally Ill (CAMI)*** is an instrument for the systematic description of the attitudes of the community towards mentally ill people [36], which consists of 40 items divided into four dimensions (Authoritarianism; Benevolence; Community mental health ideology and Social restrictiveness). Only the “Authoritarianism” 10-item dimension in the Spanish version will be used [37]. Five of the 10 items are expressed as positive and five as negative. The score for each subscale is the sum of the positive items, and the reverse of the negative items. All items are scored on an ordinal scale (5–1), respectively, ranging from 10 to 50 for each factor. Higher scores mean greater agreement with engaging in the stated attitude.

### Others outcomes

The secondary outcome of the study is the acceptability of intervention, to measure the degree of receptivity by the students towards the proposed interventions. Other outcomes of the study are the mental health symptoms and emotional wellbeing, intention to change about a mental health problem, bullying and cyber-bullying behaviours, health-related quality of life (HRQoL), help-seeking behaviours and use of treatment.

**Acceptability and satisfaction of interventions:** these variables will be assessed by a short questionnaire specially developed for this study. It consists of six items which integrate the following dimensions: 1) interesting; 2) useful; 3) practical; 4) duration; 5) resolution of doubts and 6) activity recommendations. In addition, we will use one open question to express possible proposals, claims and/or comments.

### Mental health symptoms and emotional well-being

***Strengths and Difficulties Scale (SDQ)*** (Spanish version) [38–40]. The SDQ consists of 24 items which generate scores along five subscales: emotional symptoms, conduct problems, hyperactivity-inattention, peer problems, and prosocial behaviour (positive mental health). Each item is rated 0, 1 or 2 points in accordance with being “absolutely true”, “somewhat true” or “not true”. The score is inverted in those items whose presence indicates positive features. The total score ranges from 0 to 50. It is suggested that individuals with scores above the cut-off (depending on each study population) should be evaluated more closely due to the increased risk of any alteration in mental health. The SDQ has been validated for use with adolescents aged 11–16 with a Cronbach’s alpha of 0.82 for the total difficulties scale [40, 41].

### Intentions to change

***States of Change Scale (SCS)*** (Spanish version). This is based on the Transtheoretical Change Model developed by James C. DiClemente O. Prochaska and Carlo [42, 43] to assess the degree of intention, disposition or attitude towards change. This instrument contains a total of five items which measure the process of evolutionary change in the following levels: (a) Precontemplation; (b) Contemplation; (c) Preparation for action; (d) Action; and e) Maintenance. The scale used is a Likert scale from 1 to 5 following the order of evolution of the items.

### Bullying and cyber-bullying behaviours

Four yes/no questions were specially developed for the purposes of this study to ask whether a person has been a victim or a perpetrator of a bullying and/or a cyberbullying incident.

### Health-related quality of life

The ***EuroQol 5D-5 L questionnaire (EQ-5D-5 L)*** [44, 45] is composed of two sections: the EQ-5D-5 L descriptive EQ system and the Visual Analogue Scale (EQ VAS). The descriptive system comprises five dimensions: mobility, self-care, usual activities, pain/discomfort, anxiety/depression; and each dimension has five levels: “no problems”, “slight problems”, “moderate problems”, “severe problems”, and “extreme problems”. The user response results in a 1-digit number expressing the level selected for that dimension in a Likert scale from 1 to 5, respectively. The EQ VAS records the respondent’s self-rated health on a visual analogue scale with endpoints labelled ‘the best health you can imagine’ that carries a value of 100, and ‘the worst health you can imagine’ that carries a value of 0.

### Help-seeking and use of treatment

The instrument selected consists of two parts: (i) the first question asks whether the person has received psychological and/or medicinal treatment for an MH problem at any time in her/his life; and (ii) the second part includes the first item from the Spanish version of the ***General Help-Seeking Questionnaire (GHSQ)*** for measuring help-seeking behaviour in adolescents [46, 47]. The GHSQ asks participants to respond to each problem-type by rating their help-seeking intentions on a 7-point scale ranging from 1 (“extremely unlikely”) to 7 (“extremely likely”) for each help source option including “no one.” The sum of the points gives a total score ranging from 5 to 35. Higher scores indicate higher help-seeking intentions. Additionally, we added an econometric questionnaire of 8 items on the use of services for the purpose of the cost-effectiveness study.

**Health Benefits Questionnaire:** 17-item questionnaire to include information about: a) family: parental age, studies and occupation; number of brothers/sisters and their ages; b) academic scope: years in the same school, repeated school years, average grade, number of failed subjects and number of written warnings; and c) health: difficulty sleeping and general state of health.

### Statistical analysis

To analyse the effectiveness of the EspaiJove.net intervention, an experimental design will be created to evaluate the programme, randomised by clusters (schools) and with a control group on the waiting list. The statistical analyses to be performed will be as follows. A description of the sociodemographic variables of the sample at baseline will be done through a univariate analysis of the selected variables from the total sample and stratified by type of intervention. Then, to evaluate the effectiveness of all programmes and to achieve statistical significance for all outcomes after the intervention, an unadjusted bivariate primary analysis will be performed. Data scores after the intervention will be analysed with ANOVA, assuming that the baseline data will be similar due to the randomisation process across groups. Finally, a multivariate and adjusted analysis, as necessary, will be employed to evaluate the effectiveness of those programmes so as to achieve statistical significance after the intervention. Data will be analysed with Generalised Equation Estimates (GEE) to assess the efficacy of the intervention. This statistical model is appropriate for longitudinal clustering analyses since this study design (schools) will allow us to consider the individual data correlation within the same cluster group. Analyses will be based on an intention-to-treat analysis.

We will also examine relative and absolute risk reduction, (RRR) and (ARR), respectively. Additionally, to analyse the acceptability and satisfaction of each programme, a descriptive analysis will be performed to observe the percentage of acceptability and satisfaction. Statistical analyses will be performed using the SPSS [48].

## Discussion

The aim of this paper is to describe the cRCT regarding a universal mental health literacy intervention called “EspaiJove.net” designed to promote mental health knowledge, increase help-seeking, reduce the stigma associated with mental illness, and prevent mental disorders in Spanish school settings.

Among the interventions to promote mental health and prevent mental disorders, MHL has played an important role, given that it helps young people to recognise, manage and seek appropriate help when a mental disorder develops, and it can also reduce the stigma associated with mental health.

The *EspaiJove.net* has the potential to provide a valid, new and useful school-based intervention for adolescents in Spanish settings aimed at preventing mental disorders. The contents of the intervention include useful knowledge about mental health, such as definitions of mental health and mental disorders, recognition of mental disorders, healthy behaviours and risk behaviours and bullying/cyber-bullying behaviours, as well as information about the most prevalent mental disorders during adolescence. Furthermore, this study assesses the magnitude of the stigma associated with a delay in help-seeking and the use of treatment.

Some limitations of the study protocol could be as follows. Firstly, the possible loss of missing subjects during the follow-up where there may be a possible decrease in the statistical power and selection bias. These will be minimised with successive reminders to schools, tutors/teachers, and students. Additionally, school settings promote higher retention rates of participants. The intention-to-treat imputation method will be used to analyse the missing subjects, and socio-demographic comparison analyses will be performed among missing and included subjects to search for group differences. Secondly, the selection of the study sample is limited to Barcelona city which may affect the generalisation of the results among the rest of the Spanish population.

Our study has a number of strengths including the extensive external validity of the study despite it being a cRCT. The study is conducted as close as possible to the actual conditions of the school setting. In the event of the EspaiJove.net programme being effective, it will be implemented in other secondary schools within Barcelona city in Catalonia in the future. This project will also evaluate the impact of the EspaiJove.net programme in other health outcomes, such as the intention to change, bullying and cyberbullying and HRQoL, which previous MHL interventions did not evaluate.

From our best knowledge, this is the first study to evaluate the long-term effectiveness of a MHL universal program in Spain. Results would be informative for efforts to prevent mental disorders and promote mental wellbeing in secondary school students.

## Additional file

Additional file 1: SPIRIT checklist. (DOC 124 kb)

## Abbreviations

CAMI: Community Attitudes toward the Mentally Ill
cRCT: Clustered Randomised Controlled Trial
EMHLT: EspaiJove.net Mental Health Literacy Test
MD: Mental Disorders
MH: Mental Health
MHL + SR: Mental Health Literacy plus Stigma Reduction
MHL: Mental Health Literacy
RIBS: Reported and Intended Behaviour Scale
SDQ: Strengths and Difficulties Scale
SP: Sensitivity Programme
